# A contemporary update on cancer and takotsubo syndrome

**DOI:** 10.3389/fcvm.2023.1301383

**Published:** 2024-01-08

**Authors:** Giacomo Tini, Luca Arcari, Raffaella Mistrulli, Federico Follesa, Alessandro Cianca, Matteo Sclafani, Giuliano Tocci, Paolo Spallarossa, Allegra Battistoni, Luca Cacciotti, Beatrice Musumeci, Emanuele Barbato

**Affiliations:** ^1^ Department of Clinical and Molecular Medicine, Sapienza University of Rome, Rome, Italy; ^2^ Cardiology Unit, Madre Giuseppina Vannini Hospital, Rome, Italy; ^3^ Department of Clinical, Internal, Anesthesiology and Cardiovascular Sciences, Sapienza University of Rome, Policlinico Umberto I, Rome, Italy; ^4^ Cardiovascular Disease Unit, IRCCS Ospedale Policlinico San Martino—IRCCS Italian Cardiology Network, Genova, Italy

**Keywords:** takotsubo syndrome, cancer, cardiotoxicity, anticancer treatments, Cardio-Oncology

## Abstract

Takotsubo syndrome (TTS) is characterized by a transient left ventricular systolic dysfunction, burdened by significant acute and long-term mortality and morbidity. The prognosis of TTS, especially in the long-term, is influenced by both non-cardiovascular (non-CV) and CV comorbidities, among which cancer is one of the most common. The presence of a malignancy is proven to be associated with higher mortality in TTS. Moreover, a number of anticancer treatments has been reported to possibly cause TTS as a form of cardiotoxicity, even though clearcut associations are lacking. The aim of this narrative review is to sum up contemporary knowledge on the association of cancer and TTS, addressing unmet needs and practical implications. The importance of a close collaboration between cardiologists and oncologists is herein highlighted, both to allow an adequate management of the acute TTS phase, and to actively and safely return to the oncologic management once the acute setting is resolved.

## Why talking about cancer in TTS

Takotsubo syndrome (TTS) is an acute heart failure (HF) condition, characterized by transient left ventricular (LV) systolic dysfunction (1). Alongside the classic “apical ballooning” presentation with mid-to-apical LV akinesia and basal iperkinesia (2), some “atypical” variants have been described: mid-ventricular, basal, and focal (3–5). The clinical presentation of TTS frequently resembles acute myocardial infarction (AMI), with chest pain, electrocardiographic (ECG) abnormalities and increased troponin values. Despite specific non-invasive diagnostic criteria have been proposed (6, 7), a final diagnosis of TTS is typically confirmed after coronary angiography excludes obstructive coronary artery disease as the underlying cause of the regional LV wall motion abnormalities (1, 2, 6). TTS pathophysiology has not been completely disentangled: a multifactorial genesis is likely to be accounted for its development, including catecholamine surge (i.e., an excessive sympathetic activation due to stressors), brain-heart axis alterations, acquired and genetic predisposition (1, 2, 8). At the clinical level, an identifiable trigger is often found as a precipitating factor, either as emotional or physical (i.e., surgery, infection).

Contrary to what initially thought (9–11), TTS is not a benign condition. In the acute phase it can be associated with a significant in-hospital mortality and morbidity. In the long-term, mortality risk has been shown to be similar to that of AMI (1, 12). Notably, this excess in mortality is mostly due to non-cardiovascular (non-CV) causes (2, 13–15). Indeed, the clinical course of TTS is significantly influenced by both non-CV and CV comorbidities. These are not only “contributors” of the clinical picture of vulnerable HF patients typical of TTS, but, most importantly, are prognostic factors (8, 12, 14, 16). Accordingly, TTS secondary to an emotional trigger has a relatively good prognosis, while physically triggered TTS has higher prevalence of non-CV comorbidities (17) and therefore is associated with worse outcomes (11, 17).

Cancer is one of the most common comorbidities in TTS, and a well-defined predictor of mortality (2, 12, 18). It is interesting to note that cancer can underlie both types of triggers, impacting on the emotional-psychological state and causing several physical pathological alterations (i.e., oxidative stress, inflammatory milieu, altered metabolism) (19). Moreover, a number of anticancer treatments have been reported to possibly cause TTS as a form of cardiotoxicity (20, 21). In the broader setting of HF, cancer has received large attention because of both the possible shared mechanisms and risk factors between CV diseases and malignancies, and its impact on tolerability of CV therapies and on prognosis (22, 23).

In this era of precision medicine, during which great improvements in CV and oncologic treatments and better prognosis have been recorded, an integrated approach caring for patients’ bidirectional needs appears fundamental (24, 25). In the case of a comorbid HF *and* oncologic patient, as each disease influences the management of the other, cooperation of clinicians must be pursued. However, the scientific community mostly focused on prevalence and prognostic impact of cancer in TTS. Reports of TTS in cancer patients are anectodical (i.e., case reports) or retrieved from large databases. The two aspects (cancer in TTS vs. TTS in cancer) are almost always kept separate, and data on optimal management of these cases are lacking. The aim of this narrative review is to sum up contemporary knowledge on these issues, focusing on unmet needs and practical implications.

## Impact of cancer on TTS prognosis

A systematic revision of 1,109 TTS cases published in 2014 found a 10% mean prevalence of cancer in TTS, ranging from 4% to 29% (16). Similar frequencies were found in subsequent meta-analyses (18, 26, 27). In the three largest international TTS registries, cancer prevalence ranges from about 12% to 17% (17, 28, 29). Overall, cancer in TTS appears more common as compared to the general population, as well as to patients with AMI and HF (27, 30–33). Table 1 shows prevalence of cancer in the main TTS registries published to date. Notably, the definition of cancer may vary from one study to another (i.e., any cancer vs. active cancer).

**Table 1:** Prevalence of cancer in the main TTS registries published to date.

Study	TTS patients in the cohort	Patients with cancer (*n*, %)
Arcari et al.,—GEIST (34)	2,138	312 (14.6%)
Cammann et al.,—InterTAK (28)	2,402	396 (16.5%)
Nuñez-Gil et al.,—RETAKO (29)	1,097	129 (11.8%)
Yerasi et al.,—US Nationwide Readmissions Database (35)	28,079	1,890 (6.7%)
Kim et al., (36)	265	70 (26%)
Zaghlol et al., (37)	318	81 (25.4%)
Joy et al.,—US National Inpatient Sample (38)	1,22,855	8,089 (6.6%)
Sattler et al., (39)	138	17 (12.3%)
Möller et al., (40)	286	53 (18.5%)
Lee et al., (41)	128	45 (35.2%)
Girardey et al., (42)	154	44 (28.5%)
Isogai et al.,—Japanese Diagnosis Procedure Combination database (43)	3,719	291 (7.8%)
Song et al., (44)	137	18 (13%)
Parodi et al., (45)	116	11 (9%)

Non-CV and CV comorbidities are the main determinants of outcome in TTS, especially in the long-term (12). Among these, cancer has been evaluated (specifically or among other variables) in several studies, and always resulted as a predictor of mortality at follow-up. In an analysis from the Mayo Clinic, cancer was the single leading cause of death in a cohort of 265 TTS patients (36). Some reports also highlighted the association of cancer with worse in-hospital outcome, further emphasizing how the presence of a malignancy reflects a particularly vulnerable status. Table 2 reports the main studies specifically evaluating the impact of cancer on TTS prognosis. Notably, these studies—with diverse follow-up times—mostly evaluated all-cause mortality or all-cause adverse events. Indeed, CV mortality does not appear to be influenced by cancer (40). Only two studies showed an association between cancer and both all-cause and CV mortality (42, 47).

**Table 2:** Main studies evaluating the impact of cancer on TTS prognosis.

Study	Prevalence and type of cancer	Follow-up duration	Findings
Tini et al., (24)	13% (active)	2.7 years	Cancer associated with all-cause mortality (OR 3.1)
Genc et al.,—meta-analysis (46)	6.8%	-	Cancer associated with higher in-hospital or 28-day risk of death (OR 2.3)
Guo et al.,—meta-analysis (27)	-	-	Cancer associated with all-cause mortality (RR 2.2)
Núñez-Gil et al., (29)	11.8% (any)	27.5 months	Cancer associated with all-cause mortality (OR 1.7)
Nguyen et al., (47)	16.8% (any)	4.1 years	Cancer associated with all-cause mortality (HR 2.4), but not at multivariate analysis
Cammann et al., (48)	16.6% (any)	5 years	Cancer associated with all-cause mortality (HR 1.8)
Brunetti et al.,—meta-analysis (18)	6.7%	-	Cancer associated with in-hospital (RR 2.1) and follow-up risk of adverse events (RR 3.3)
Zaghlol et al., (37)	25.4% (any)	-	Cancer associated with in-hospital cardiac arrests (OR 9.3)
Kim et al., (36)	26% (any)	5.8 years	Cancer associated with all-cause mortality (HR 1.9)
Joy et al., (38)	6.6% (any)	-	Cancer associated with all-cause mortality (OR 3.4)
Möller et al., (40)	20% (any)	4 years	Cancer associated with all-cause mortality (HR 1.6)
Sattler et al., (39)	14% (any)	4.2 years	Cancer associated with follow-up risk of adverse events (HR 2.4)
Girardey et al., (42)	28.5% (any)	≈1 year	Cancer associated with all-cause mortality (HR 2.6)

CV management of an oncologic subject suffering TTS may be challenging. Pharmacologic treatment of TTS involves HF drugs (in particular beta blockers and renin-angiotensin system inhibitors), which could be associated with better outcomes (12, 49), but may also be poorly tolerated in cancer (21, 22, 50, 51). Moreover, it has been reported that cancer patients are frequently under-treated in the CV setting (21, 25, 52–54). On the other hand, from an oncologic perspective, chronic CV comorbidities or acute events (as TTS) may be perceived as debilitating when pertaining a cancer patient, possibly impacting delivery of optimal treatments (22, 24, 50). This latter aspect may be alarming when one considers how mortality in TTS is typically comorbidity-related (i.e., cancer-related), and thus it might be enhanced by an oncologic undertreatment due to the acute CV event. Nonetheless, the only analysis to date, though from a small cohort, comparing TTS patients with cancer to a non-TTS matched cancer counterpart, showed similar prognosis in the two groups, suggesting that mortality is unrelated to the TTS event (24).

## TTS as a form of cardiotoxicity due to anticancer drugs

CV adverse events related to anticancer treatments are defined as “cardiotoxicity”. This term represents a heterogeneous group of conditions, including LV dysfunction and HF, venous thromboembolism, arterial thrombotic events, arrhythmias, myocarditis (20, 55). TTS has been included among this long list of adverse events, due to a range of cancer therapies. The most commonly associated agents are pyrimidines (19, 20), but TTS in patients treated with anti-VEGF agents, cisplatinum, gemcitabine, trastuzumab or anthracyclines has also been reported. Nevertheless, a specific underlying mechanism by which each of such treatments might cause TTS is not known. Moreover, there is a paucity of data concerning dosages and time frame in which TTS develops (19).

In a report from the US National Inpatient Sample database including 1,067,977 patients hospitalized with chemotherapy-related admissions between 2010 and 2014, the incidence of TTS was 53 per 100,000 cases (56). Those developing TTS were more commonly females and had a significantly higher risk of in-hospital mortality as compared to the group that did not develop TTS. This study did not report information regarding the type of treatments received by patients developing TTS.

In a recent meta-summary of 41 case reports of TTS occurring during diverse anticancer treatments, 5-fluorouracil was the most commonly reported drug, in more than one third of cases (57). Patients usually developed TTS a few days after the delivery of the anticancer treatment (median time: 2 days). TTS caused cardiogenic shock in one quarter of cases; at follow-up a complete recovery of LV function was reported in 89% of cases. In another study evaluating 27 cases, 5-fluorouracil was again the anticancer treatment most commonly associated with TTS (41% of cases) (58). TTS tended to develop shortly after the administration of chemotherapy, even hours, and most commonly occurred in women.

The pathogenetic mechanism of pyrimidine-associated TTS is unknown, likewise the overall mechanisms of pyrimidine-associated cardiotoxicity have not been completely elucidated. Coronary vasospasm and microvascular dysfunction have been proposed as putative mechanisms contributing to its pathophysiology, overall and specifically for TTS (59–62). However, few case reports described absence of overt or induced vasospasm during acute episodes of pyrimidine-associated angina or TTS (60, 63). In other cases, either the presence of not hemodynamically significant coronary artery disease was reported, or a history of previous coronary artery disease resulted as a predictor of cardiotoxicity development. In such scenarios plaque instability or vasospasm causing a narrowing of the coronary lumen on top of a pre-existing plaque might not be excluded (19, 64, 65). Finally, recent findings suggest that the main mechanisms of pyrimidine-associated cardiotoxicity are direct vascular (endothelial) and myocardial damage (59, 61). In summary, despite many reports suggest pyrimidines to possibly cause TTS, this association remains hypothetical, as “pure” coronary events cannot be ruled out with certainty.

Discontinuation of the “culprit” treatment is suggested when TTS occurs during anticancer therapy (20). This holds true for pyrimidines; however, a successful rechallenge after a cardiotoxic event has been reported in some cases (62).

In recent years the indications for immune checkpoint inhibitors (ICI) therapy widened, and concurrently there was an exponential increase in reports of ICI-associated cardiotoxicity (66). Outside of the well-known issue of myocarditis, other cardiac adverse events due to ICI have been reported, including atrial fibrillation, myocardial infarction, pericardial effusion and TTS. Through the World Health Organization (WHO) global database of individual case safety reports VigiBase®, 13 reports of TTS in patients receiving ICI drugs emerged (all with ICI monotherapy). Data on clinical evolution was available in eight of the reported cases: one patient died, four recovered LV systolic function, while three others did not (67). ICI-related TTS was observed in seven other case reports (68–74).

As for pyrimidines, a clear mechanism underlying the development of TTS in ICI is unknown. In several cases, an interesting overlap between TTS and myocarditis was described (75). In a registry of 30 ICI-related myocarditis, a “TTS–like appearance” occurred in 14% (76). Bearing in mind that myocarditis is a well-known differential diagnosis of TTS (1), the association of ICI with TTS remains difficult to be clearly confirmed.

After a cardiac adverse event, ICI are usually discontinued, and rechallenge is not recommended. However, this approach is strongly suggested for myocarditis, but there are no clear indications for other types of cardiotoxicities (20, 77). Re-challenge of ICI after cardiotoxicity have been proposed, but few data are available in this regard (78).

TTS due to anticancer treatments is a relatively rare event. However, it may have significant implications. Its occurrence may prompt discontinuation of the oncologic treatment, which may be even definitively halted, given the absence of clear recommendations. However, understanding the true association of a given anticancer treatment with a TTS event may be challenging, considering that the oncologic status itself may be causing TTS, or that TTS might not be diagnosed with certainty (i.e., difficulties in performing a thorough differential diagnosis with other conditions). Indeed, a clearcut association of anticancer drugs with TTS is frequently absent, and in some case reports, LV systolic function recovery is not always present. These diagnostic and nosologic issues appear difficult to overcome. Nevertheless, in clinical practice, the most important aspect remains that of an optimal management of the patient. A close collaboration between cardiologists and oncologists is needed, both to deliver an adequate CV management in the acute phase, and to actively and safely return to the oncologic management once the acute HF setting is overcome. Two aspects are very important and must be considered. First, as oncologists have the task to decide if, when and how to restart an anticancer treatment, cardiologists should be supportive in giving their perspective. Even in the case of a supposed association between TTS and the anticancer treatment, TTS recurrencies are uncommon (79) and should not be the only reason for a treatment to be permanently discontinued (80). Secondly, as for other CV risk factors or prior events, rather than to evaluate their presence vs. absence, it should be considered whether a condition is stable and well-treated, and which was its clinical course (54, 81). This is very important in Cardio-Oncology, where specific CV *comorbidities* may significantly influence the oncologic management. In a certain way, after the acute TTS phase, the cardiologic perspective should be of reassurance and cooperation toward the oncologist and the oncologic management (24).

## Practical implications and conclusions

The presence of cancer in a TTS patient should be recognized by clinicians as a proxy of a frail status, predisposing to a worse outcome, especially in the long-term. Although this concept might appear easy to understand, it is not as straightforward to translate it into clinical practice and holds significant implications.

Following the resolution of the acute phase, the management of TTS entails a shift from a single specialist (i.e., cardiologist) approach, to a comprehensive, inter- and multi-disciplinary management (82). An integrated treatment of each comorbidity appears fundamental, yet in everyday clinical practice this may not be easy to be pursued. It is worth noting that the high prevalence of cancer in TTS has prompted some Authors to suggest an oncologic screening of these patients (48). However, no clear data have been provided in this regard, and to date, there is no clear indication for performing such a screening.

When TTS occurs in a patient with a malignancy actively treated, the goal of the management after the acute CV phase should be to allow a safe return-to-therapy in the oncologic setting. A close follow-up in the first 3 to 6 months after the acute event, a period during which LV systolic function and ECG completely recover in most cases, must be performed, and a tailored HF therapy (i.e., according to patients' tolerability) must be delivered. During this period, a strict interaction with the oncologist should be maintained to allow initiation or re-initiation of the anticancer treatment. Given the paucity of data on this regard, and the uncertainties on clearcut association of specific anticancer treatments with TTS occurrence, we suggest that (re-)initiation of the oncologic therapy is discussed case-by-case in a multi-disciplinary setting. This means that not only aspects related to the TTS events should be considered, but also those related to the oncologic treatment. Clinicians should face the following questions: has the anticancer treatment possibly caused TTS? What was the clinical course of TTS and has LV systolic function recovered in the short-term? What is the oncologic risk of the patient? Is it feasible for the patient to pause the anticancer treatment (either it is associated with TTS or not) and to possibly re-start it in a few months or is it preferable not to stop the oncologic treatment? Coming up with the best solution to all these issues should be the priority of patient's management once the acute TTS phase has resolved. In most cases, and when the anticancer treatment may be safely paused, initiation or re-initiation of the oncologic therapy may be done after 3 months from the TTS event. With careful management, a re-challenge of the anticancer treatment used before the TTS event may be tested. Once again, the oncologic aspect is crucial in this decision (i.e., is that the best treatment for the patient and the one with the highest expected efficacy or are there any comparable solutions?). On the contrary, observing an uncomplete recovery of LV systolic function should raise concern. Since this is quite unusual in TTS (1), suspicion that myocardial injury (and not TTS) has occurred should be considered. Therefore, in such cases, a modification of the oncologic treatment is more reasonable.

In conclusion, a “holistic” Cardio-Oncology approach (25), dedicated to a multi-disciplinary patient's management (and not limited to the evaluation of cardiotoxicity) appears as the best method to care for TTS patients with cancer.
